# Wood for Finishing Fillings

**Published:** 1861-10

**Authors:** 


### WOOD FOR FINISHING FILLINGS.

At the suggestion of Dr. Allport, we have recently been using small rods of box wood; such as used by watchmakers, for a vehicle for polishing material, such as pumice, etc., for finishing fillings and for wedging between the teeth while filling. The wood is of a very fine and close texture, and yet soft; it has not the hard, unyielding texture of hickory, and yet it is sufficiently hard for the purpose.

The wood is obtainable in bundles of rods wherever watchmakers' tools are sold. It may be cut into any desired form for polishing. The depots will furnish it. T.

